# An Improved Experimental Model of Hemorrhoids in Rats: Evaluation of Antihemorrhoidal Activity of an Herbal Formulation

**DOI:** 10.1155/2014/530931

**Published:** 2014-03-11

**Authors:** Mohammed Azeemuddin, Gollapalle Lakshminarayanashastry Viswanatha, Mohamed Rafiq, Agadi HireMath Thippeswamy, Mirza Rizwan Baig, Kethaganahalli Jayaramaiah Kavya, Pralhad Sadashiv Patki, Ramakrishnan Shyam

**Affiliations:** Department of Pharmacology, R&D Center, The Himalaya Drug Company, Makali, Bangalore, Karnataka 562 162, India

## Abstract

*Objective*. To improve the existing experimental model of croton oil-induced hemorrhoids in rats by using Evans Blue (EB) dye extravasation technique. Further, an herbal formulation (Pilex) was evaluated for its antihemorrhoidal activity in this model. *Methods*. Two sets of experiments were carried out: first to improve the experimental model and to validate the same using Pilex and second to evaluate the effect of Pilex on cytoarchitecture of rectoanal tissue in croton oil-induced hemorrhoids. In both sets, hemorrhoids were induced to all the animals, except normal controls, by applying croton oil via rectoanal region and the effect of Pilex ointment (PO), Pilex granules (PG), and combination of PG and PO was evaluated. In the first set, extravasation of EB dye, TNF-**α**, IL-6, and rectoanal coefficient (RAC) was determined. In the second set, severity of score, RAC, and histopathology were evaluated. *Results*. The elevated levels of TNF-**α**, IL-6, and extravasations of EB dye were decreased with the Pilex treatment. The cytoarchitecture of rectoanal portion of the animals treated with Pilex was near to normal. *Conclusion*. The improved experimental model of hemorrhoid is useful in quantifying the inflammatory exudates and extent of inflammation. In this improved experimental model Pilex showed antihemorrhoidal activity, which further validates its clinical usage.

## 1. Introduction

Today, millions of people are suffering from hemorrhoids and it is more prone as you grow older, and it is becoming a major medical and socioeconomic problem. There are various factors responsible for hemorrhoids like constipation, sedentary life style, pregnancy, low fiber diet, obesity, and so forth. Usually, hemorrhoids develop due to increase in pressure on the veins of the pelvic and rectal region, which causes abnormal dilatation and distortion of the vascular channel, leading to the extravasation of blood around the perianal and anal vein, which results in rectal bleeding [1–3]. The experimental model reported by Nishiki et al. in 1988 [4] to evaluate the antihemorrhoidal activity in rats assesses qualitative/semiquantitative aspects associated with hemorrhoids. Thus, to overcome this limitation, a new experimental model has been developed to quantify the extravasation of inflammatory exudates and markers involved in hemorrhoids.

Treatment of hemorrhoids in modern medicine is still in infancy. Given the fact that there is no specific drug to treat hemorrhoids, extensive research is ongoing in the field of Ayurveda for utilizing the natural sources for treating hemorrhoids. Pilex, a polyherbal proprietary formulation of The Himalaya Drug Company (Bangalore, India), is used clinically over the years for the treatment of hemorrhoids and other related conditions. Several reports are available indicating the beneficial effects of Pilex [5–8].

Pilex granules constitute a mixture of extracts of Guggulu (*Balsamodendron mukul*), Shilajeet (purified), Nimba seeds (*Melia azadirachta*), Daruharidra (*Berberis aristata*), Amalaki (*Emblica officinalis*), Haritaki (*Terminalia chebula*), Vibhitaki (*Terminalia bellirica*), Aragvadha (*Cassia fistula*), Kanchanara (*Bauhinia variegata*), and Nagkesara (*Mesua ferrea*). Pilex ointment constitutes a mixture of extracts of Lajjalu (*Mimosa pudica*), Bhringaraja (*Eclipta alba*), Nirgundi (*Vitex negundo*), Zergul (*Calendula officinalis*), Karpoora (*Cinnamomum camphora*), Tankana, and Yashada bhasma. Pilex is approved by the Government of India's Drug Regulatory Authority (Department of Ayush, Ministry of Health and Family Welfare). It also conforms the principles of Good Manufacturing Practice (GMP) and Good Laboratory Practice (GLP). Its botanical identification, quality parameters, and ayurvedic criteria are complied with the international guidelines and pharmacopoeial standards [9, 10].

With the above background, the present study was aimed to improve the experimental model of croton oil-induced hemorrhoids in rats and to validate the same using a known herbal formulation (Pilex) for treatment of hemorrhoids.

## 2. Materials and Methods

### 2.1. Drugs and Chemicals

Croton oil (Sigma Aldrich, St. Louis, USA), Evans Blue (Loba Chemie, Bombay, India), Pilex (The Himalaya Drug Company, Bangalore, India), and all the other chemicals used in the experiments were of analytical grade from reputed suppliers.

### 2.2. Experimental Animals

Inbred male Wistar rats (200–250 g) that were housed in standard conditions of temperature (22 ± 3°C), relative humidity (55 ± 5%), and light (12 h light-dark cycle) before and during the study were included in the experiment. They were fed with standard pellet diet (Provimi Animal Nutrition India Pvt. Ltd., Bangalore, India) and water* ad libitum*. All the experimental protocols were approved by the Institutional Animal Ethics Committee (IAEC) of The Himalaya Drug Company. The animals received humane care as per the guidelines prescribed by Committee for the Purpose of Control and Supervision of Experiments on Animals (CPCSEA), The Ministry of Environment and Forests, Government of India.

### 2.3. Experimental Protocols

Two sets of experiments were carried out. The first set was used to improve an existing experimental model of hemorrhoids (mentioned by Nishiki et al. [4]) and to validate the same by using Pilex granules (PG), Pilex ointment (PO), and a combination of both. The protocol was designed to quantify the extent of plasma exudation and to determine the levels of inflammatory cytokines such as TNF-*α* and IL-6 associated with hemorrhoids. In the second set, the effect of PG, PO, and a combination of both were further confirmed by determining the rectoanal coefficient (RAC), severity score, and the histopathological evaluation [11–16].

### 2.4. Improvement of Existing Experimental Model of Hemorrhoids and Evaluation of Pilex (First Set)

Male Wistar rats (200–250 g) were randomly divided into 5 groups (G-1–G-5) of 8 each (*n* = 8). G-1 and G-2 animals received water (10 mL/kg) and served as normal and positive controls, respectively; G-3 animals received PG (400 mg/kg, PO); G-4 animals received PO (200 mg/animal, intrarectal (ir)); G-5 animals received combination of PG (400 mg/kg, PO) along with PO (200 mg/animal, ir). All the groups received the assigned treatments once daily for 5 days after the induction of hemorrhoids.

Hemorrhoids were induced to all the groups, except normal control group, by applying croton oil preparation (deionized water, pyridine, diethyl ether, and 6% croton oil in diethyl ether in the ratio of 1 : 4 : 5 : 10). Followed by an overnight fasting, sterile cotton swabs (4 mm diameter) soaked in 100 *μ*L of croton oil preparation were inserted into the anus (rectoanal portion, 20 mm from anal opening) of all the study animals and kept for 10 seconds. A linear development of edema was observed up to 7 to 8 hours after the croton oil application. Quantitative evaluation of croton oil-induced plasma exudation in the rectoanal tissue of rats was determined by estimating the quantity of Evans Blue (EB) dye. EB dye (30 mg/kg) was injected through the tail veins of the animals, 30 min before the application of croton oil preparation to induce hemorrhoids. Twenty-four hours after the induction, animals of the respective groups were treated for 5 days. On the fifth day, 1 hour after the relevant treatment, blood samples were collected from retroorbital sinus for estimating the levels of TNF-*α* and IL-6. All animals were euthanized by exsanguination under deep isoflurane anesthesia; their rectoanal tissues (20 mm in length) were isolated and weighed and the EB dye present in the tissue was extracted using 1 mL of formamide. The absorbance of the sample was recorded using Synergy HT (multimode microplate reader, BioTek) at 620 nm and quantified using standard curve of EB dye [13–16].

### 2.5. Evaluation of Pilex against Croton Oil-Induced Histological Changes in Rats (Second Set)

Male Wistar rats (200–250 g) were randomized based on their body weights and were divided into 7 groups (G-1–G-7), with each group consisting of 8 animals (*n* = 8). G-1 and G-2 animals received water (10 mL/kg) and served as normal and positive controls, respectively; G-3 animals received PO (200 mg/animal, ir); G-4 and G-5 animals received PG (200 and 400 mg/kg b.wt., PO, resp.); animals in G-6 and G-7 received a combination of PG (200 and 400 mg/kg b.wt., PO, resp.) and PO (200 mg/animal, ir).

Hemorrhoids were induced to all the groups, except normal control group, by applying croton oil preparation. Twenty-four hours after induction, all the animals were subjected to respective treatment as assigned to the groups (G-1–G-7) once daily for 5 days. On the fifth day, 1 hour after the treatment, all the animals were euthanized by exsanguination under deep isoflurane anesthesia and rectoanal tissues (20 mm in length) were isolated. They were evaluated for the severity score, weighed, and fixed in 10% neutral buffered formalin solution for histological examination. The RAC was calculated using the formula

(1) Rectoanal  coefficient =Weight  of  rectoanal  tissue  (mg)Body  weight  (g).

Histological observation of the rectoanal tissue was made to note the appearance of inflammatory cells, congestion, hemorrhage, vasodilatation, and medium to high degrees of necrosis [11, 12].

### 2.6. Statistical Analysis

The results of the study were expressed as mean ± SEM and analyzed by one-way ANOVA with Dunnett's posttest using GraphPad Prism version 4.01 for Windows, GraphPad Software, San Diego, California, USA, and *P* value less than 0.05 was considered statistically significant.

## 3. Results

**Figure 1:**
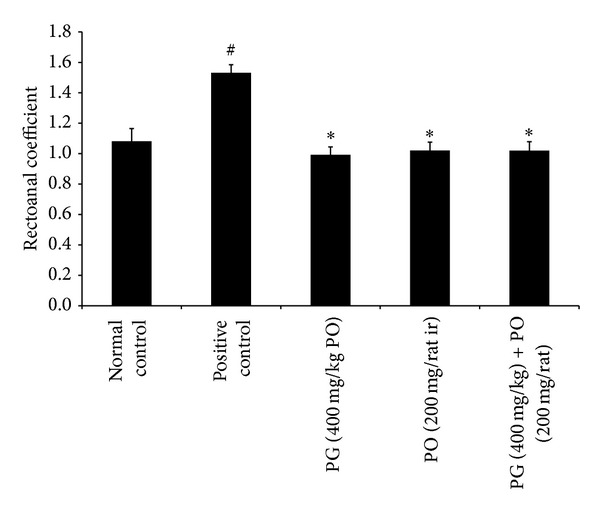
Effect of Pilex on rectoanal coefficient. All the values are expressed as mean ± SEM. The mean values of all the groups were compared by one-way ANOVA followed by Dunnett's posttest. ^#^ *P* < 0.01 compared to normal control; *P < 0.05 compared to positive control. PG: Pilex granules; PO: Pilex ointment.

**Figure 2:**
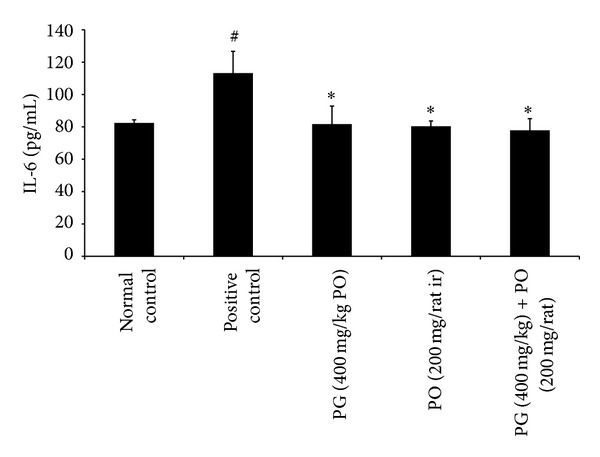
Effect of Pilex on serum IL-6 in rat model of croton oil-induced hemorrhoids. All the values are expressed as mean ± SEM. The mean values of all the groups were compared by one-way ANOVA followed by Dunnett's posttest. ^#^ *P* < 0.01 compared to normal control; *P < 0.05 compared to positive control. PG: Pilex granules; PO: Pilex ointment.

**Figure 3:**
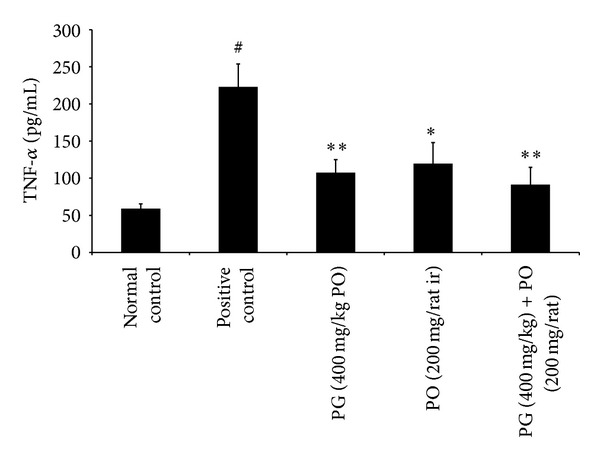
Effect of Pilex on serum TNF-*α* in rat model of croton oil-induced hemorrhoids. All the values are expressed as mean ± SEM. The mean values of all the groups were compared by one-way ANOVA followed by Dunnett's posttest. ^#^ *P* < 0.01 compared to normal control; *P < 0.05 and **P < 0.01 compared to positive control. PG: Pilex granules; PO: Pilex ointment.

**Figure 4:**
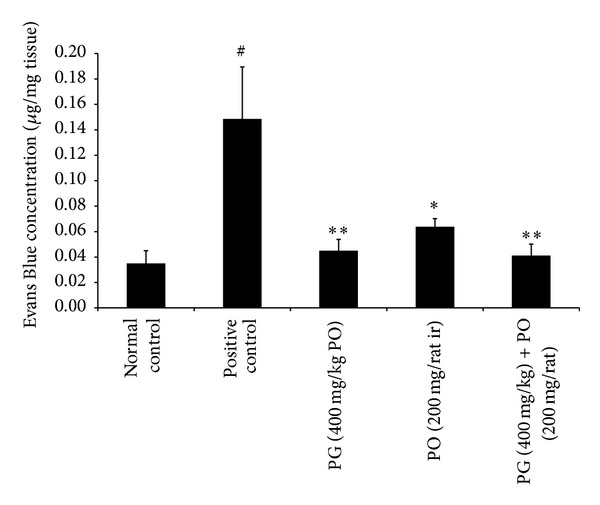
Effect of Pilex on Evans Blue dye extravasation in the rectoanal tissue of rat. All the values are expressed as mean ± SEM. The mean values of all the groups were compared by one-way ANOVA followed by Dunnett's posttest. ^#^ *P* < 0.01 compared to normal control: *P < 0.05 and **P < 0.01 compared to positive control. PG: Pilex granules; PO: Pilex ointment.

In the first set of experiment, the application of croton oil to rectoanal portion of rats induced significant increase in the RAC (*P* < 0.01) and serum level of proinflammatory cytokines such as IL-6 (*P* < 0.01) and TNF-*α* (*P* < 0.01); it also showed a significant increase in the extravasation of EB dye (*P* < 0.01) in rectoanal portion compared to normal control animals. However, the treatment with PG (400 mg/kg; *P* < 0.05), PO (200 mg/rat; *P* < 0.05), and combination of PG (400 mg/kg) with PO (200 mg/animal) (*P* < 0.05) has maintained the RAC and IL-6 levels near to normal control group (Figures 1 and 2). Moreover, the effect of PO (200 mg/animal; *P* < 0.05), PG (400 mg/kg; *P* < 0.01), and combination of PG (400 mg/kg) and PO (200 mg/animal; *P* < 0.01) was highly significant against elevated levels of TNF-*α* when compared to positive control group (Figure 3). In line with all these, the PG (400 mg/kg, PO), PO (200 mg/animal, ir), and combination of PG (400 mg/kg, PO) with PO (200 mg/animal, ir) have offered a significant protection against increased extravasation of EB dye due to croton oil application (Figure 4).

**Figure 5:**
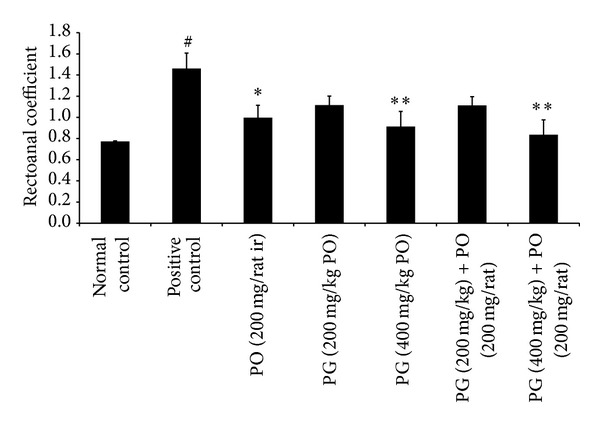
Effect of Pilex on rectoanal coefficient in rat model of croton oil-induced hemorrhoids. All the values are expressed as mean ± SEM. The mean values of all the groups were compared by one-way ANOVA followed by Dunnett's posttest. ^#^ *P* < 0.01 compared to normal control: *P < 0.05 and **P < 0.01 compared to positive control. PG: Pilex granules; PO: Pilex ointment.

In the second set of experiment, application of croton oil to rectoanal portion of rats induced significant alterations in RAC, severity score, and histopathological findings. The RAC of normal control and positive control groups was found to be 0.77 ± 0.006 and 1.46 ± 0.14 (*P* < 0.01), respectively. The positive control group showed 1.89fold higher RAC value when compared to normal control. Interestingly, treatment with PG (400 mg/kg, PO; *P* < 0.01), PO (200 mg/animal, ir; *P* < 0.05), and a combination of PG (400 mg/kg, PO) with PO (200 mg/animal, ir; (*P* < 0.01) alleviated the conditions. The reversal was found to be dose-dependent in case of PG and PG with PO in combination (Figure 5).

**Figure 6:**
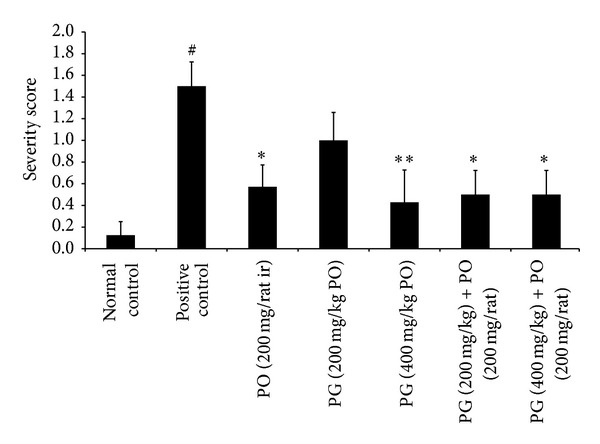
Effect of Pilex on severity scores of rectoanal tissue. All the values are expressed as mean ± SEM. The mean values of all the groups were compared by one-way ANOVA followed by Dunnett's posttest. ^#^ *P* < 0.01 compared to normal control; *P < 0.05 and **P < 0.01 compared to positive control. PG: Pilex granules; PO: Pilex ointment.

**Figure 7:**
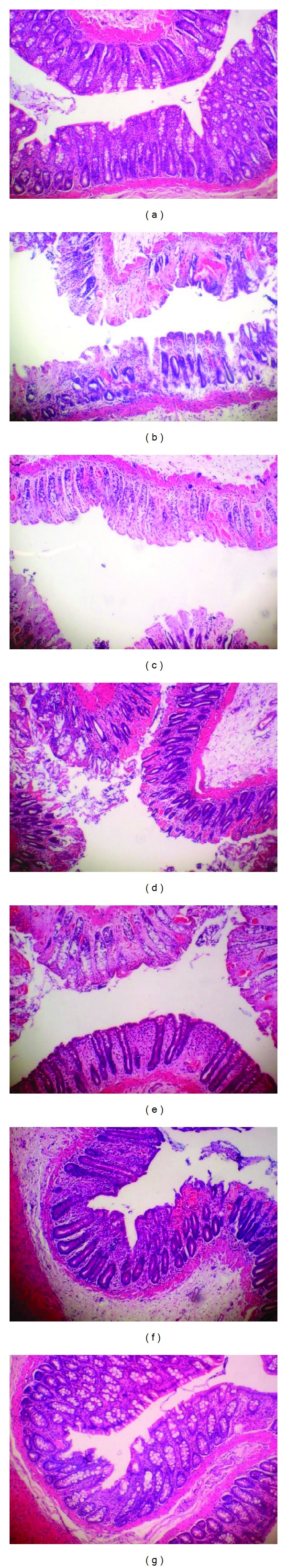
Effect of Pilex on histology of rectoanal tissue in a rat model of croton oil-induced hemorrhoids. Recto anal sections of rats: (a) section showing normal appearance and architecture in normal control group; (b) marked to severe inflammation, congestion, haemorrhage, dilatation of blood vessels, degeneration, and necrosis can be observed in the section of untreated positive control group; (c) section of rat treated with Pilex ointment (200 mg/rat) showing mild inflammation, marked congestion, degeneration, and necrosis; (d) and (e) sections of rat treated with Pilex granules (200 & 400 mg/kg p.o.) showing minimal inflammation, congestion, haemorrhage, dilatation of blood vessels, degeneration, and necrosis; (f) and (g) sections of rat treated with Pilex granules (200 & 400 mg/kg) along with Pilex ointment showing near normal architecture (H&E × 200).

Additionally, the isolated rectoanal tissue was visually observed and scored based on the severity; the findings showed a severity score of 0.12 ± 0.35 in normal control group and 1.50 ± 0.22 (*P* < 0.01) in the positive control group, which was statistically significant compared to normal control. Interestingly, the treatment with PO (200 mg/animal, ir; *P* < 0.05), PG (400 mg/kg, PO; *P* < 0.01), and combination of PG (200 mg/kg, PO) with PO (200 mg/animal, ir; *P* < 0.05) and PG (400 mg/kg, PO) with PO (200 mg/animal, ir; *P* < 0.05) has significantly ameliorated the croton oil-induced recto anal damage compared to positive untreated control (Figure 6). These findings were further supported by histopathological examination wherein the animals treated with PO (200 mg/animal, ir), PG (200 & 400 mg/kg PO), and combination of PG (200 & 400 mg/kg, PO) with PO (200 mg/animal, ir) showed significant reversal in the severity of inflammation, vasodilatation in the rectoanal portion, and presence of inflammatory cells (such as leukocytes, neutrophils, and macrophages) along with hypertrophy of the mucosal cells and hemorrhagic spots (caused due to croton oil-induced hemorrhoids). The normal control animals showed normal cytoarchitecture of the rectoanal region (Figure 7 and Table 1).

## 4. Discussion

It is well proved that hemorrhoids are a pathological condition, which is characterized by a severe vasodilatation at the rectoanal region, which leads to inflammation of the surrounding tissues, thus further leading to secondary complications such as extravasation of fluid into interstitial space mainly due to increased vascular permeability and migration of large quantity of inflammatory cells (granulocytes and monocytes). In the present study, croton oil has been used as inducer/phlogistic agent to induce experimental hemorrhoids. Croton oil causes inflammation due to the release of soluble factors involving inflammatory lipid metabolites (prostaglandins, leukotrienes, and lipoxins), kinins (bradykinins and chemokines), nitric oxide, and cytokines (TNF-*α* and IL-6). These factors, alone and/or in combination, regulate the activation of resident cells (fibroblasts, endothelial cells, macrophages, and mast cells) and newly recruited inflammatory cells (monocytes, lymphocytes, neutrophils, and eosinophils) leading to systemic response to inflammation (fever and cachexia) [15]. In the present set of experimental investigation, EB dye extravasation test, cytokines estimation (quantification of soluble inflammatory factors), and histopathological evaluations (presence of inflammatory cells) were performed to link the series of inflammatory reactions involved in the development of hemorrhoids due to croton oil application [17].

The results of present studies showed severe extravasation of EB dye and increased levels of proinflammatory cytokines (TNF-*α* and IL-6) in experimental animals. These pathological changes were supported by histological changes of the rectoanal portion exhibiting severe vasodilatation and inflammatory cells infiltration, along with hypertrophy of the mucosal cells and hemorrhagic spots. The sham/normal control group showed normal cytoarchitecture of the rectoanal region.

Interestingly, 5 days of treatment with PG, PO, and their combination have significantly ameliorated the inflammatory hallmarks of croton oil-induced hemorrhoids in rats compared to positive control. This may be due to the potent anti-inflammatory activity of the herbs present in the tested formulation, such as,* M azadirachta, E officinalis*,* T chebula*, and* B aristata* [18–22].

There was a dose-dependent decrease in RAC and pathological abnormalities were observed in groups treated with PG and combination of PG with PO, which may be due to the presence of herbs such as* T chebula*,* M pudica, *and* A indica *in Pilex and are traditionally used in the management of hemorrhoids [23]. However, the precise molecular mechanism behind the antihemorrhoidal activity of Pilex needs to be explored.

## 5. Conclusions

The findings of the present study suggest that the improved experimental model described in the present work has advantage of being sensitive compared to previously described models in the literature, as this improved model has a provision for quantifying the inflammatory exudates in the induced experimental hemorrhoids. Also, it was found that, among the herbal combinations evaluated in the improved model, PG, PO, and combination of PG with PO ameliorated the croton oil-induced hemorrhoids in rats. Notably, the combination of PG with PO was found to be more potent when compared to their* per se* treated groups in the second set of experiment, where RAC and histopathological findings were evaluated.

## Figures and Tables

**Table 1:** Histopathological evaluation of rectoanal portion of rats.

Lesions	Normal control	Positive control	PO (200 mg/animal, ir)	PG (200 mg/kg, PO)	PG (400 mg/kg, PO)	PG + PO (200 mg/kg, PO + 200 mg/animal, ir)	PG + PO (400 mg/kg, PO + 200 mg/animal, ir)
Inflammation	0.5 ± 0.3	3.25 ± 0.3	3.00 ± 0.3	3.00 ± 0.0	2.75 ± 0.5	3.00 ± 0.00	1.00 ± 0.57**
Congestion/hemorrhage	0.0 ± 0.0	3.00 ± 0.0	2.00 ± 0.9	3.00 ± 0.0	3.00 ± 0.0	2.50 ± 0.95	0.75 ± 0.47*
Dilatation of blood vessels	0.5 ± 0.3	3.00 ± 0.0	1.50 ± 0.9	2.75 ± 0.6	2.33 ± 0.7	2.75 ± 0.62	0.75 ± 0.47*
Necrosis/loss of superficial epithelium	0.0 ± 0.0	4.00 ± 0.0	2.50 ± 0.9	4.00 ± 0.0	3.00 ± 0.0	3.50 ± 0.25	1.75 ± 0.48*

Severity score: NAD: 0 (no abnormality detected); minimal: 1 (very small amount of changes ≤10 %); mild: 2 (lesion is easily identified but limited severity 11–25%); moderate: 3 (lesion is predominant 26–75%); severe: 4 (the degree of changes is 76–100% or great enough in intensity or extent to expect significant tissue or organ dysfunction).

Values are expressed as mean ± SE.*P < 0.05, **P < 0.01, versus positive control.

PG: Pilex granules; PO: Pilex ointment.
